# Nutritional Profiling and the Value of Processing By-Products from Gilthead Sea Bream (*Sparus aurata*)

**DOI:** 10.3390/md18020101

**Published:** 2020-02-04

**Authors:** Mirian Pateiro, Paulo E. S. Munekata, Rubén Domínguez, Min Wang, Francisco J. Barba, Roberto Bermúdez, José M. Lorenzo

**Affiliations:** 1 Centro Tecnológico de la Carne de Galicia, Rúa Galicia No 4, Parque Tecnológico de Galicia, San Cibrao das Viñas, 32900 Ourense, Spain; mirianpateiro@ceteca.net (M.P.); paulosichetti@ceteca.net (P.E.S.M.); rubendominguez@ceteca.net (R.D.); robertobermudez@ceteca.net (R.B.); 2 Preventive Medicine and Public Health, Food Science, Toxicology and Forensic Medicine Department, Faculty of Pharmacy, Universitat de València, Avda. Vicent Andrés Estellés, s/n, 46100 Burjassot, València, Spain; minwang@alumni.uv.es (M.W.); francisco.barba@uv.es (F.J.B.)

**Keywords:** fish discards, valuable compounds, amino acids, fatty acid profile, mineral composition

## Abstract

Fish processing industries generate a large volume of discards. In order to fulfil with the principles of a sustainable circular economy, it is necessary to maintain aquaculture by-products in the food chain through the production of high-value biomolecules that can be used as novel ingredients. In this study, we try to give value to the gilthead sea bream by-products, evaluating the composition and the nutritional value of the muscle and six discards commonly obtained from the fish processing industry (fishbone, gills, guts, heads, liver, and skin), which represent ≈ 61% of the whole fish. Significant differences were detected among muscle and by-products for fatty acid and amino acid profile, as well as mineral content. The discards studied were rich in protein (10%–25%), showing skin and fishbone to have the highest contents. The amino acid profile reflected the high quality of its protein, with 41%–49% being essential amino acids—lysine, leucine, and arginine were the most abundant amino acids. Guts, liver, and skin were the fattiest by-products (25%–35%). High contents of polyunsaturated fatty acids (PUFAs) (31%–34%), *n*-3 fatty acids (12%–14%), and eicosapentaenoic acid (EPA) + docosahexaenoic acid (DHA) (6%–8%) characterized these discards. The head displayed by far the highest ash content (9.14%), which was reflected in the mineral content, especially in calcium and phosphorous. These results revealed that gilthead sea bream by-products can be used as source of value-added products such as protein, oils, and mineral supplements.

## 1. Introduction

Nowadays, aquaculture is probably the fastest growing food production sector [1]. In 2016, global aquaculture production accounted for almost 50% of the world’s fish products destined for food, including 80.0 million tonnes of food fish and 30.1 million tonnes of aquatic plants. Regarding food fish outputs, 54.1 million tonnes were from finfish, 17.1 million tonnes were from molluscs, 7.9 million tonnes were from crustaceans, and 938,500 tonnes were from other aquatic animals [2]. China is by far the largest world producer at 15.3 million tonnes, followed by other Asiatic countries such as Indonesia, India, Japan, and Viet Nam. In Europe, Norway and Spain top the list at 2.03 and 0.91 million tonnes of production, respectively.

However, this large production volume also involves the generation of by-products that are not intended for human consumption. In 2016 approximately 37,900 tonnes of non-food products were obtained from fish processing industries [2]. These aquaculture by-products include backbones, belly flaps, fish fins, gills, heads, liver, roe, skin, viscera, and meat adhered to the bones [3]. The ratio between food fish and by-product varies by fishing fish size and species, season, and zone [4]. Thus, bones can account for percentages of 9%–15% of whole fish, while the head comprises 9%–12%, viscera 12%–18%, skin 1%–3%, and scales 5% [5].

On the other hand, fish one of the most nutritious animal-derived foods [6]. It contains high-quality proteins, balanced essential amino acids, high levels of fat-soluble vitamins (A and D), and essential macro and micro minerals (iodine, magnesium, phosphorus, and selenium) [7]. Fish nutrient composition depends on fish species, age, gender, health, nutritional status, and time of the year [8]. It is characterized by 15%–30% proteins, 0%–25% lipids, and 50%–80% moisture. Thus, depending on their fat content there are lean fish such as cod and hake with compositions of 20% protein, 80% water, and rather low lipid levels (0.5%–3%); fatty fish, such as mackerel and salmon, have the same protein contents but higher fat content (10%–18%) and therefore lower water contents (62%–70%) [9]; and gilthead sea bream, sea bass, and trout are classified as semi-fatty fish.

The healthy compounds present in fish composition are also part of its by-products. Therefore, it is necessary to analyze these by-products, especially regarding their nutritional properties. This will allow us to characterize more in detail these wastes and the active substances which could be present, and thus determine the value of these products [10]. The bioactive compounds, which can be extracted from foods or foods by-products, are nutritional elements that are usually present in small quantities, and are able to regulate metabolic functions, leading to beneficial effects [11]. Therefore, the extraction of bioactive compounds is one of the most feasible options for utilization of by-products [12]. In fact, a great number of bioactive compounds can be obtained from aquaculture by-products [3,13,14], allowing an efficient use of fish by-products, transforming them into ingredients that can be incorporated into feed, food, or other high-value products [15,16]. The use of fish by-products in animal feeds (flours and oils) is common today [17,18]. Moreover, these phytochemicals, especially polyunsaturated fatty acids (PUFAs), minerals, proteins, and peptides, have several nutritional benefits, which together with their biological activities could give rise to a beneficial effect on human health [19,20,21]. Among their fatty acids, high levels of long chain *n*-3 fatty acids stand out, associated with reduction of the risk of cardiovascular diseases in humans [22,23].

Gilthead sea bream (*Sparus aurata*) is one of the most important marine finfish species reared in Europe, and is mainly produced in the Mediterranean and Northern European countries [24,25], where it represents 4.1% relative production of the primary aquaculture species. Aquaculture allows for the production of gilthead sea bream at a low cost [26]. Traditionally, it was reared extensively in coastal lagoons and saltwater ponds in almost all Mediterranean countries. Its characteristics related to a high survival rate and feeding habits makes it suitable for extensive aquaculture in the Mediterranean. In the 1980s, the intensive systems that have been evolving to this day began to develop, successfully achieving the artificial reproduction of this species whereby the production of juveniles increases, with intensive captive breeding both in ponds and in floating cages [27]. The rapid development of the production in floating cages of culture in the sea has allowed the development of the sector, increasing the production of this species.

Little information exists about the chemical composition of by-products from Mediterranean species. Therefore, the aim of this study was characterized the nutritional value of several by-products (fishbone, gills, guts, head, liver, and skin) obtained from the processing of gilthead sea bream, comparing their composition with those presented in muscle, the portion most commonly marketed. 

## 2. Results

### 2.1. Fish Dissection Characteristics of Gilthead Sea Bream By-Products

The dissection percentages of gilthead sea bream are shown in Table 1. Sea bream pieces used to carry out the study had mean weights of 0.484 kg and mean lengths of 0.27 m. The outcomes of fish dissection suggested that gilthead sea bream provided high yields of noble parts, since muscle represented the predominant cut, satisfying the industry and consumer demands. More specifically, as mentioned previously, muscle was the cut that represented the higher percentage with respect to the whole piece, followed by head, fishbone, and skin which showed values above 10% (34.12%, 21.53%, 14.57%, and 10.25% for muscle, head, fishbone, and skin, respectively). Next in importance are guts, with mean values of 5.04%. Liver, sometimes included with guts as viscera, represented a mean percentage of 1.31%.

In the dissection, fins were also taken into account. Anal, caudal, dorsal, pectoral, and pelvic fins were obtained. They represented 1.75% of the whole piece, with the caudal fin being predominant, followed by dorsal, pectoral, and pelvic fins (33.10%, 24.54%, 21.69%, and 13.70% of total fins, respectively). Finally, the anal fin supposed percentages lower than 10% of total fins.

### 2.2. Nutritional Characterization of Gilthead Sea Bream By-Products

#### 2.2.1. Proximate Composition of Gilthead Sea Bream By-Products

The chemical composition of the by-products analysed is presented in Table 2. The type of by-product studied showed a significant effect (*p* < 0.001) on the moisture, fat, protein and ash contents of sea bream. Moisture values ranged between 45.11% and 69.07%, being the muscle the part that had higher percentages. Regarding fat content, guts, liver, and skin were the portions that achieved the highest percentages (34.11%, 25.76%, and 26.78%, respectively).

Concerning protein contents, fillet and skin showed the highest contents (higher than 20%), followed by fishbone, gills, and head (16.39%, 13.92%, and 12.91%, respectively). Finally, ash content also showed significant differences (*p* < 0.001) depending on the portion studied. Head, gills, and fishbone where the parts that had higher contents, achieving values greater than 6% of the proximate composition (9.14%, 6.45%, and 6.23%, respectively).

#### 2.2.2. Fatty Acid Profile of Gilthead Sea Bream By-Products

The fatty acid composition of the different fish by-products of sea bream is summarized in Table 3. The results obtained showed that the studied by-product significantly (*p* < 0.001) affected the fatty acid profile. Monounsaturated fatty acids (MUFAs) were the predominant fatty acids (44.1–47.6 g/100 g of total fatty acids), followed by polyunsaturated fatty acids (PUFAs) (31.2–34.0 g/100 g of total fatty acids) and saturated fatty acids (SFAs) (19.7–20.9 g/100 g of total fatty acids). Regarding individual fatty acids, C18:1*n*-9 was the major fatty acid (33.0–35.9 g/100 g of total fatty acids), followed by C16:0 (13.3–14.1 g/100 g of total fatty acids), and C18:2*n*-6 (16.6–19.6 g/100 g of total fatty acids).

Concerning MUFAs, the predominant fatty acid was oleic acid, showing levels of around 76% of the total MUFA. The highest values were obtained in liver and gills, while fishbone and head showed the lowest values. The type of cut also affected the amounts of C16:1*n*-7, C17:1*n*-7, C18:1*n*-7, C20:1*n*-9, C22:1*n*-9, and C24:1*n*-9; however, these fatty acids were a minority, with amounts below 5.0 g/100 g of total fatty acids.

With respect to PUFAs, it is important to highlight the concentrations of *n*-3 PUFAs (11.9–14.2 g/100 g of total fatty acids), and long chain *n*-3 PUFAs (6.9–9.8 g/100 g of total fatty acids), with muscle and head being the samples that showed the highest contents, respectively. These differences were mainly due to the variations in C18:3*n*-3 (3.9–4.7 g/100 g of total fatty acids), C20:5*n*-3 (1.8–2.8 g/100 g of total fatty acids; eicosapentaenoic acid, EPA), C22:5*n*-3 (1.4–2.0 1.8–2.8 g/100 g of total fatty acids; docosapentaenoic acid, DPA), and C22:6*n*-3 (3.5–5.2 1.8–2.8 g/100 g of total fatty acids; docosahexaenoic acid, DHA) contents.

In regard to SFA, the concentrations found were higher in fishbone, liver, and head than in the other fish parts. These differences could be mainly due to the highest amounts of C18:0 (2.7–4.5 g/100 g of total fatty acids; *p* < 0.001) and C16:0 (13.3–14.1 g/100 g of total fatty acids; *p* < 0.01). In contrast, guts and skin samples showed the lowest concentrations of these fatty acids (19.8 and 19.7 g SFA/100 g of total fatty acids, respectively). 

In order to verify how the type of by-product affects the nutritional quality of fat, the nutritional indices of DHA/EPA, *n*-6/*n*-3, and PUFA/SFA were calculated. The type of waste has a significant effect (*p* < 0.001) on these ratios. Liver had the highest DHA/EPA ratios, with mean values of 2.6 vs. 1.7 obtained in fishbone. The *n*-6/*n*-3 ratio was the lowest in liver, fillet and head, while guts showed the highest values (1.40 vs. 1.75, respectively). Within PUFAs/SFAs, gills displayed the lowest ratio and fillet the highest (1.55 vs. 1.70, respectively).

#### 2.2.3. Amino Acid Profile of Gilthead Sea Bream By-Products

The amino acid composition of the different fish by-products of sea bream are shown in Table 4. The fish waste analysed exhibited a significant effect (*p* < 0.001) on the amino acid profile. The results showed that the predominant amino acids were non-essential, with the exception of muscle that had higher contents of essential amino acids. Regarding the by-product studied, fillet and skin showed the highest contents, gills displayed intermediate values, and guts and liver obtained the lowest amounts of total amino acids (TAAs).

Regarding the essential amino acids fraction, lysine was the most abundant, followed by leucine and arginine, representing together about 47% of essential amino acids and 22% of TAAs. Gills and liver were the organs that showed the lowest percentages. Methionine was the amino acid that presented the lowest values (range from not detected values in gills and guts to 346.5 mg/100 g of tissue, representing around 2% of essential amino acids). On the other hand, glutamic acid, aspartic acid, and glycine were the most abundant in the non-essential fraction, representing together around 61% of non-essential amino acids and 33% of TAAs, with liver being the organ that showed the lowest percentages. In this case, the lowest values were observed for tyrosine (range from 219.1 to 587.8 mg/100 g of tissue), representing around 6% of total non-essential amino acids; muscle and skin displayed the highest values (587.8 and 532.2 mg/100 g of tissue, respectively), fishbone showed intermediate values (455.1 mg/100 g of tissue), and values lower than 300.0 mg/100 g of tissue were observed in the rest of by-products.

Regarding by-product studied, except for the head, the most abundant amino acid was glutamic acid (1001.5–2541.7 mg/100 g of tissue; around 14% of TAAs). In fillet and skin this amino acid showed values around 2500 mg/100 g, around 1000 mg/100 g in guts and liver, and 1500 mg/100 g in gills and head. From this moment, the profile of the by-products began to differentiate. Some similarities were found in the profile of by-products depending on its protein content. In muscle and skin, lysine and aspartic acid were of importance, showing similar values (around 1700 mg/100 g of tissue; around 10% of TAAs). The similarity in this profile could be due to the protein content of these parts (21.05 vs. 24.78%, respectively). There was a similar finding in fishbone and gills, where glycine and aspartic were predominant. The values found in fishbone for aspartic acid were close to those found in the aforementioned cuts. In the case of gills, the contents were two times less than those observed in fishbone (873.9 vs. 1490.5 mg/100 g of tissue for gills and fishbone, respectively). Similar values to the gill were those found in guts and liver (707.0 vs. and 580 mg/100 g of tissue, respectively).

Finally, the values of the essential/non-essential (E/NE) ratio were in the range 0.71–1.08 and were significantly (*p* < 0.001) affected by the type of by-product, with fillet being the by-product which showed the highest ratio, while gills had the lowest value.

#### 2.2.4. Protein Quality: Chemical Score of Amino Acids of Gilthead Sea Bream By-Products

The nutritional quality of protein of gilthead sea bream by-products is shown in Table 5. The results obtained showed that the by-products studied had a significant effect (*p* < 0.01) on the chemical score of the essential amino acids (CS) and the essential amino acids index (IEAA).

Histidine and threonine were the amino acids which presented the highest values of CS. In the first case, fishbone and head obtained the best scores (229.8 and 200.3%, respectively), while guts and skin displayed the worst scores for this amino acid. Fishbone showed the best results for threonine, followed closely by the gills and head. On the other hand, methionine was a limiting amino acid in all of the by-products studied, displaying values lower than 100%. In addition to this amino acid, isoleucine and leucine were also lacking in gills and guts. The different by-products studied were not limited in aromatic amino acids (Phe + Tyr) in relation to the reference protein, for which percentage varied from 127.7 to 183.5%.

Finally, statistical analysis showed significant differences (*p* < 0.001) for the IEAA index, which was highest for fishbone, followed by muscle, liver and head (151.7, 139.0, 124.0, and 123.5, respectively), while the lowest values were obtained in gills and guts (66.7 and 58.6, respectively).

#### 2.2.5. Mineral Content of Gilthead Sea Bream By-Products

The concentrations of minerals in the different by-products of sea bream are shown in Table 6. A tissue effect was also observed on the mineral content (*p* < 0.001), both in macro-minerals and micro-elements. The higher ash content of gills, head, and fishbone commented upon before was also reflected in the mineral content of the by-products studied.

Regarding macro-minerals, calcium and phosphorous was the most abundant; however, this content is highly variable and dependent on the tissue studied. In calcium, the contents were very different, with values of 2389.24, 1873.24, and 1618.83 mg/100 g in head, gills, and fishbone compared to the lower values observed in cuts such as liver and guts (12.81 and 19.44 mg/100 g, respectively). A similar behaviour was observed in phosphorous, where head, gills and fishbone showed five times higher values than the other by-products. Potassium and sodium followed the aforementioned elements in abundance. In the first case, fillet and fishbone displayed values higher than 300 mg/100 g, while gills and guts presented half of these contents (135.0 and 153.4 mg/100 g, respectively). A similar phenomenon occurred with liver and gills in sodium. Their values were above 250 mg/100 g, while skin, fillet, and fishbone showed values well below (lower than 125 mg/100 g).

Regarding micro-minerals, iron and zinc were the minerals that showed the higher contents, with values that exceeded in all cases 0.5 mg/100 g. In both cases liver was the by-product that showed the highest values (3.82 and 3.46 mg/100 g for iron and zinc, respectively), followed in importance by gills and guts with mean values of 2.12 and 1.95 mg/100 g, respectively. In contrast, the contents of copper and manganese found in the by-products studied were very low, lower than 600 µg/100 g. Liver and gills were the cuts that showed the higher amounts (536.0 and 585.1 µg/100 g for copper and manganese, respectively). In contrast, the contents of copper in the head and the amounts of zinc in fillet and skin showed trace levels.

## 3. Discussion

### 3.1. Processing Yield and Fish Dissection Characteristics of Gilthead Sea Bream

The total yields of by-products from gilthead sea bream were about 61% of the whole fish based on wet weight. This result was in agreement with those reported for different kinds of commercial fish (50%–60%) [29] and higher than those found by other authors in anchovy [30]. 

During the filleting process, a common processing step that is used in the preparation of muscle for the production of other products, large quantities of wastes are generated [31]. Fishbone and skin are included between these products, as reflected in the percentages that these products represented of the whole fish (14.6% and 10.3%, respectively). According to the results found by other authors, the highest yields among different by-products were found in head, while viscera (mainly represented by liver and guts) supposed lower percentages of the whole fish (6.35% vs. 14.4% and 8.9% for gilthead sea bream vs. salmon and anchovy, respectively) [29,30].

### 3.2. Nutritional Characterization of Gilthead Sea Bream By-Products 

#### 3.2.1. Proximate Composition of Gilthead Sea Bream By-Products

There are several factors that affect the proximate composition of fish and therefore that of their by-products. Fish species, age, gender, health, nutritional status, and time of the year are among the factors that affect the chemical composition [32]. This could be observed when the results found in the present study were compared with those found by other authors. Muscle moisture, protein, and ash contents were similar to those found by other authors in sea bream [33,34]. However, the fat contents were quite different, probably due to some of the aforementioned factors. In fact, these contents can range between 5.2% and 10.5% [35,36]. There was a similar finding when this composition was compared with other Mediterranean aqua cultured fish species like sea bass (*Dicentrarchus labrax*) and common dentex (*Dentex dentex*) [33,37]. Pleadin et al. [37] found a similar proximate composition in these three species, while Özden and Erkan [33] found that the analysis of moisture and fat displayed different compositions, with higher moisture (69.7% and 71.8% vs. 76.4% for sea bass, sea bream, and common dentex, respectively) and lower fat (6.5% and 8.1% vs. 2.3% for sea bass, sea bream, and common dentex, respectively) contents in common dentex. Significant differences were also found when these species were compared with Atlantic species like cod (*Gadus morhua*), especially regarding its high moisture and low-fat contents (81.22% and 0.67%, respectively) [38]. 

These differences increased when the muscle was compared with the by-products studied. The highest contents of protein, fat, and ash were found in skin, guts, and head, respectively. These results were in agreement with previous studies of fish processing wastes on various species such as Alaska pollock and Pacific cod [39]. According to the results found by these authors, skin displayed significantly higher protein values than muscle. This could be related to the fact that during the processing small amounts of muscle could remain in the skin, partly explaining the differences found.

Regarding ash, head and fishbone accumulated significant amounts of minerals in their composition, in accordance with other authors who claim that there are important depots of minerals in these discards [40]. However, the ash contents found in head were higher than those found in other fish processing wastes [29], and similar to those found by Suseno et al. [41] in the head of small pelagic fish.

Viscera (guts and liver) with the skin were the by-products that showed the highest fat contents. With respect to viscera, the liver composition found in the present work were similar to the results found by other authors in salmon viscera (61.2% moisture and 27.2% fat) and in other cold-water fish species such as flathead sole [29,42].

#### 3.2.2. Fatty Acid Profile of Gilthead Sea Bream By-Products

The content and the composition of specific fatty acids are important for assessing the nutritional quality of fish by-products, its suitability and its potential for incorporation of fish oil into food products [43]. To date, many studies have measured the fatty acid composition of fish muscle but little work has focused on the fatty acid profile of fish by-products, and the possibility of extracting lipids from these discards for use as functional ingredients. As with the results found by other authors, the fatty acid profile varied among species, breeding systems, and by-products [26,44]. Some authors affirmed that PUFA contents were higher than SFA and MUFA contents in spring and summer; whereas in autumn, SFA levels were higher than either PUFA or MUFA levels [44]. In our case we disagree with this statement, since the samples were captured in October and MUFAs were the predominant fatty acids. 

Although significantly different, the fatty acid profile of guts and liver was similar to those found in muscle of sea bream, which would allow its use in functional foods as a substitute of the oil obtained from edible fillet. This is in accordance with the results found by other authors in farmed Atlantic salmon (*Salmo salar* L.) viscera [45]. In accordance with the results of these authors, C16:0, C18:1*n*9, 20:5*n*3, and 22:6*n*3 are among the predominant fatty acids identified in muscle, guts, and liver. These fatty acids also appear as a majority in cut-off, liver, and viscera of cod, ling, saithe, and haddock, reporting the highest contents in liver during the autumn catch [44]. 

Regarding omega-3 fatty acids, EPA, DPA, and DHA are essential fatty acids for human health that cannot be synthesised by the human body, so they have to be sourced from food [46]. In this regard, fish is the main source and it has been reported to contain higher amounts of these fatty acids [47]. In the present study, sea bream viscera contained important contents of EPA (1.83 and 1.91 g/100 g in guts and liver, respectively) and DHA (3.51 and 3.98 g/100 g in guts and liver), but these contents were lower than those found in fatty fish species like tuna and salmon. Ferdosh et al. [48] found that DHA was the major PUFA identified in tuna, with contents of 17.0%–19.9% in head, 15.7%–17.3% in skin, and 14.3–16.1% in viscera. In Alaska pink salmon (*Oncorhynchus gorbuscha*), contents of 7.6% in head and 10.9% in viscera were found for EPA, and percentages of 11.8% and 17.3% in head and viscera, respectively, for DHA [49]. Lower contents were found by He et al. [29] in the viscera of Atlantic salmon (*Salmo salar*), with mean values of 5.8% and 7.3% for EPA and DHA, respectively. Our values also contrast with those found in cod offal and liver, which displayed values of 8.9% and 7.7% for EPA, and 13.3% and 11.4% for DHA, respectively. These values were even exceeded by those found in haddock [44]. These differences could be due to the type of fish and seasonal variations, as well as environmental and geographical changes [13].

These fatty acids have been demonstrated to have important roles in human health [23,47], reducing the risk of cardiovascular diseases. In fact, there are general recommendations for daily dietary intakes of DHA/EPA. The values found for this ratio showed higher values in muscle and liver (2.28 and 2.56, respectively). Similar values were obtained by other authors in the muscle of sea bass, sea bream, and common dentex (2.01, 2.64 and 2.66, respectively) [33], while lower ratios were found for pink salmon by-products (ratio of 1.6 in head and viscera) [49].

These values reflect that viscera (liver and guts) are an important source of oils rich in EPA and DHA, which could be used for the production of these omega-3 fatty acids [50]. In fact, livers form marine fish are usually used for the production of oils [42]. Hence, the results obtained indicated that gilthead sea bream by-products are a good source of fatty acids, especially omega-3 fatty acids.

The nutritional quality increases when the *n*-6/*n*-3 ratio decreases and when PUFA/SFA levels increase. The values of the *n*-6/*n*-3 ratio in all fish discards were lower than the recommended levels for the human diet (*n*-6/*n*-3 < 4; [51]). On the other hand, all the by-products studied exceed the typical values of the Mediterranean diet (0.5–0.7) [52] and the FAO recommendations for PUFA/SFA ratio in human diet (0.85; [51]). Liver and gills were the cuts that displayed the lowest values.

#### 3.2.3. Amino Acid Profile and Protein Quality of Gilthead Sea Bream By-Products

Fish is an important source of dietary amino acids for human to sustain an adequate protein nutrition and health. In fact, fish contains high amounts of protein and balanced proportions of all amino acids relative to human requirements [53]. Therefore, it is assumed that by-products extracted from fish would also be an important and potential source of proteins and amino acids.

Unlike the results found by other authors, significant differences (*p* < 0.001) were found in all of the amino acids identified [29,42]. Except for head, where the most abundant amino acid was glycine (13.7% of TAA), glutamic acid was the predominant amino acid (14.4% of TAA). This amino acid together with glycine was the most abundant in head, skin, and viscera obtained from Atlantic salmon, but the glycine contents were higher than those found in the present study [29].

The proline contents found in by-products were higher than those found in the muscle, with percentages above 5% vs. 3.5% of TAA content observed in muscle. The skin, fishbone, and head displayed the highest contents, with values of 948.6, 932.4, and 883.5 mg/100 g of tissue, respectively. The greatest presence of proline in these discards could be related to the higher amounts of connective tissues found in organs and bone [30,54]. In fact, this amino acid, together with alanine, glycine, and valine, represents more than 80% of the collagen and gelatin amino acid composition [13]. Hydroxyproline is another amino acid which also constitutes an important part of the composition of collagen [55]. The presence of a high percentage of these compounds has been reported to have a positive effect on the structural stability of gelatin and, therefore they have an important role in the valorisation of fish by-products through bioactive peptide production [31]. In the present study, hydroxyproline was identified in head and fishbone, with mean values of 503.7 and 393.7 mg/100 g of tissue, respectively (data not shown). Similar values to those found in fishbone were observed by Shahidi et al. [38] in cod offal (2.40 vs. 2.38 g/100 protein for gilthead sea bream fishbone and cod offal, respectively).

According to the results found by other authors for sulfur amino acids, small amounts of methionine and cysteine were found in fish discards [56]. In fact, cysteine was only quantified in fishbone and head, with values of 88.4 and 181.0 mg/100 g of tissue, respectively (data not shown).

Taurine was also identified in the by-products studied. This amino acid, present in marine organisms, is an essential nutrient that humans are not able to biosynthesize well, since it results from sulfur amino acid metabolism [57]. Some studies have been suggested that could be used as marker of seafood consumption with beneficial health effects, especially against the development of cardiovascular diseases [58]. The contents found for taurine in muscle are within the taurine levels in whole fish muscle (50–300 mg/100 g) [57]. These values are similar to those found in red sea bream (*Chrysophtys major*), and in other fish species such as Atlantic cod (*Gadus morhua*) and Atlantic salmon (*Salmo salar*), presenting values of 138, 120, and 130 mg/100 g, respectively [59,60,61]. In contrast, the values obtained in by-products were higher than those found in muscle, except for gills and liver where it was not detected. The highest values were obtained in guts (335.2 mg/100 g of tissue), followed by skin, head and fishbone with values within the range mentioned above (187.9, 166.5 and 148.7 mg/100 g of tissue, respectively).

Regarding the essential amino acids, lysine was the most abundant, followed by leucine and arginine, together representing about 47% of essential amino acids. In the present work, arginine was included in the essential amino acid fraction, since it is considered a conditionally essential amino acid [62]. Skin, liver, and head were the discards which showed the highest percentages of the aforementioned amino acids, respectively. Lower values were obtained for arginine and leucine in heads and viscera of Pacific ocean perch [42].

As commented before, fish are considered an excellent protein source, containing all essential amino acids and having high bioavailability. However, the nutritional value of a protein, referred to its “amino acid score”, is based on its composition of essential amino acids. Data on IEAA are scarce and most refer to animal production [63]. According to the results found by other authors, the CS values obtained in the muscle of gilthead sea bream were higher than 100% [64]. Therefore, there was no limiting amino acid in muscle. In the case of processing by-products, in contrast with the outcomes found by Zhong et al. [65] in carp by-products, histidine was one of the amino acids which presented the highest values of CS, while methionine was the limiting amino acid in all of the gilthead sea bream by-products studied.

The amino acid profile of the different discards of gilthead sea bream and the chemical score of their amino acids showed that its by-products had a quality protein, and therefore they are suitable candidates to produce nutritious food products or dietary protein supplements.

#### 3.2.4. Mineral Content of Gilthead Sea Bream By-Products 

Fish is considered a good source of minerals, especially calcium, magnesium, and phosphorous, but also of potassium, iron, copper, and zinc [6]. However, the differences observed between gilthead sea bream and other fish species could be due to seasonal and biological differences (species, size, age, and sex), food source, and environment factors (salinity, temperature, and contaminants) [7,66].

The contents found for these elements in muscle are within the usual values in fish, at about 10–100 mg/100 g for calcium, 200–400 mg/100 for potassium, 10–170 mg/100 g for magnesium, 200–300 mg/100 g for phosphorous, 0.3–2.8 mg/100 g for iron, 0.3–1.3 mg/100 g for zinc, and 0.1–0.2 mg/100 g for copper. When the contents of muscle were compared with those found in other species, we observed that the contents of sodium were similar to those found in sea bream and turbot, and higher than those obtained in sea bass and dentex (968.2 and 1212.3 mg/kg vs. 706.3 and 465.4 mg/kg, respectively). There was a similar finding with phosphorous, although in this case, sea bass and sea bream presented similar values (2180.2 and 2214.1 mg/kg, respectively). On the other hand, Pleadin et al. [37] found calcium contents that were double than those observed in the present study (540.2 vs. 291.1 mg/kg, respectively). Sea bream showed the lowest values for this element when it was compared with the previous species, but they were similar to those found by Petrović et al. [67].

However, it is worth highlighting the remarkable concentration of some of these minerals in the by-products studied, reflecting the higher ash content of gills, head and fishbone commented before. Despite these contents, the studies on the use of these minerals are scarce [68]. According to Gencby et al. [30], significant differences were found between muscle and the different by-products. There are several factors that could justify these variations, such as the metabolism of fish, the rate in which minerals are available in body water and the ability of the fish to absorb them [69,70].

Macro-mineral content was highly variable and depended on the tissue studied. Fishbone, gills, and head exceeded the normal values obtained for macro-minerals in muscle. Calcium and phosphorous were the most abundant minerals. The high calcium values found in fishbone reflect that this by-product is an important source of this element, an important and an essential mineral for human health and wellbeing, and can be used as a potential food supplement [71]. However, as mentioned earlier, its bioavailability has not been extensively investigated. The same behaviour was observed in Alaska pollock, and Pacific cod and pink salmon by-products [56]; however, the contents found were below than those observed in other fish species such as Black Sea anchovy (*Engraulis encrasicholus*), Atlantic Salmon (*Salmo salar*), or Pacific Ocean perch (*Sebastes alutus*) [29,30,42]. A similar trend was observed in phosphorous, where head, gills, and fishbone showed higher values. Unlike in vegetables, in fish by-products phosphorous is completely available, since it is not contained in phytic acid [72]. Potassium and sodium followed the aforementioned elements in abundance. The values obtained for sodium contrast with the fact that fish is considered a poor source of sodium. However, the values found for gills, guts, head, and liver were higher than the usual contents in the muscle (20–140 mg/100 g), with values in all cases greater than 150 mg/100 g.

Regarding micro-minerals, iron and zinc were the minerals that showed the higher contents, being liver the by-product which showed the highest values, following in importance by gills and guts. The obtained values confirm that fish by-products could be considered a suitable source of iron, since they even increase the content contributed by the fish (1–2 mg/100 g) [73]. In contrast, copper and manganese contents were very low (below 600 µg/100 g), where liver and gills were the cuts that showed the highest amounts. In contrast, the copper contents in head and the amounts of zinc in muscle and skin showed trace levels. These results were in accordance with the levels of manganese and zinc found in pollock, cod, and salmon processing by-products, which were the lowest in muscle, while the content of iron was higher in viscera [56]. High contents of copper, iron, and zinc were also found in the viscera of Pacific Ocean perch (*Sebastes alutus*), but these values were higher than those found in the present work (7.8, 93.7, and 308.0 mg/kg vs. 5.4, 34.6, and 38.2 mg/kg for viscera and liver of perch and sea bream, respectively) [42]. 

According to these results, gilthead sea bream fishbone, gills and head can be considered as good sources of calcium and phosphorous, while liver had important amounts of copper, iron, and zinc.

## 4. Materials and Methods

### 4.1. Experimental Design and Fish Sampling

**Figure 1:**
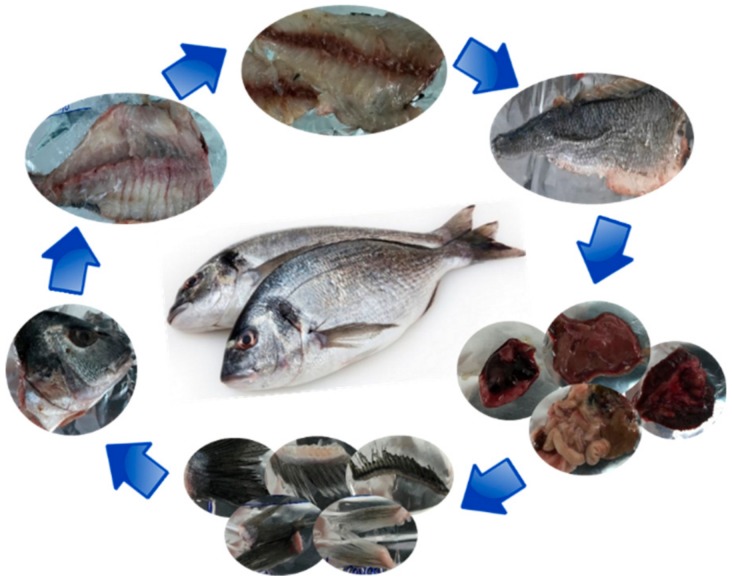
Dissection of gilthead sea bream.

A total of 10 pieces from male sea bream aged under two years were used to carry out the study. The animals, collected in a local market, were captured in the east of Spain. The sampling was done for five weeks, acquiring two pieces every week. Then, the samples were transported to the laboratories of the Centro Tecnológico de la Carne de Galicia, where the pieces were weighed and dissected. The weight and length of fish were determined. After that, fish portions (fins, fishbone, gills, guts, head, heart, liver, muscle, skin, and others) were obtained as shown in Figure 1. Once the different fish parts were obtained, we proceeded to the nutritional characterization of the selected portions (fishbone, gills, guts, head, liver, muscle, and skin). The samples were homogenized and frozen at −80 °C until their analysis. The proximate composition, fatty acid and amino acid profile, and mineral content were determined.

### 4.2. Chemical Composition

The chemical analysis (moisture, protein, and ash content) was assessed according to the ISO recommended standards 1442:1997 [74], 937:1978 [75], and 936:1998 [76]. Fat was extracted in an Ankom XT10 extractor (ANKOM Technology Corp., Macedon, NY, USA) following the A.O.C.S. Official Procedure Am 5-04 [77].

### 4.3. Fatty Acid Profile

For fatty acid analysis, total fat was extracted from 10 g of sample [78]. The fatty acids were transesterified according to the procedure of Domínguez et al. [79] with modifications: For the fatty acid transesterification, 20 milligrams of extracted fat dissolved in 1 mL of toluene were mixed with 2 mL of a sodium methoxide (0.5 N) solution, vortexed during 10 s, and allowed to stand for 15 min at room temperature. Then, 4 mL of a H_2_SO_4_ solution (10% of H_2_SO_4_ in methanol) were added, vortexed for a few seconds, and vortexed again before adding 2 mL of saturated sodium bicarbonate solution. For the extraction of fatty acid methyl esters, 1 mL of hexane was added to the samples and vortexed for 10 seconds, and the organic phase was then transferred to an appropriate GC vial.

Separation and quantification of fatty acid methyl esters (FAMEs) were carried out using a gas chromatograph (GC-Agilent 7890B, Agilent Technologies, Santa Clara, CA, USA) equipped with a flame ionization detector (FID) and PAL RTC-120 autosampler. One microliter of sample was injected in split mode (1:50). The injector was maintained at 250 °C and 64.2 mL/min of total flow. For the separation of FAMEs, a DB-23 fused silica capillary column (60 m, 0.25-mm i.d., 0.25-μm film thickness; Agilent Technologies, Santa Clara, CA, USA) was used. Chromatographic conditions were as follows: initial oven temperature of 50 °C (held for 1 min), first ramp at 25 °C/min to 175 °C, second ramp at 4 °C/min to 230 °C (held for 5 min), and third ramp at 4 °C/min to a final temperature of 240 °C (held for 2.75 min). Helium was used as carrier gas at a constant flow-rate of 1.2 mL/min, with the column head pressure set at 22.9 psi. The FID detector was maintained at 280 °C, while the operational flows were set as 40 mL/min of H_2_, 450 mL/min of air, and 30 mL/min of makeup flow. The total time for chromatographic analysis was 30 minutes. Data acquisition and equipment control was carried out using the software MassHunter GC/MS Acquisition B.07.05.2479 (Agilent Technologies, Santa Clara, CA, USA), while the data analysis was carried out with the software MassHunter Quantitative Analysis B.07.01. Individual FAMEs were identified by comparing their retention times with those of authenticated standards (FAME Mix-37 components; docosapentaenoic acid, *trans*-vaccenic acid, *cis*-vaccenic acid, and conjugated linoleic acid - CLA) and the results were expressed as g/100 g of total fatty acids identified.

The total saturated fatty acid (SFA), monounsaturated fatty acid (MUFA), and polyunsaturated fatty acid (PUFA) contents were calculated. To assess the nutritional properties of fat the DHA/EPA, *n*-6/*n*-3, and PUFA/SFA ratios were determined.

### 4.4. Amino Acid Profile

The hydrolysis of the protein, derivatization and identification of amino acids was carried out following the procedure and using the equipment described by Domínguez et al. [80]. Completion of hydrolysis of any tyrosine phenol modification was accelerated by heating for 10 min at 55 °C. Moreover, tryptophan determination was not possible because acidic hydrolysis transforms it into ammonium. Data regarding amino acid composition were expressed in mg/100 g of tissue.

### 4.5. Protein Quality: Chemical Score of Amino Acids

Once the amount of amino acids in the different muscles was determined, the chemical score of the essential amino acids (CS) was calculated in relation to the reference on pattern protein proposed by FAO/WHO/UNU [28] applying the following equation:

$$CS=\frac{g\;\mathrm{EAA}\;\mathrm{in}\;\mathrm{tested}\;\mathrm{protein}}{g\;\mathrm{EAA}\;\mathrm{in}\;\mathrm{pattern}\;\mathrm{protein}}\times 100$$

The essential amino acid index (IEAA) value was also calculated applying the following equation described by Shahidi and Synowiecki [81]:

$$IEAA=100\times \sqrt[{n}]{\left(\frac{a}{ap}\times \frac{b}{bp}\times \frac{c}{cp}\times \ldots \frac{j}{jp}\right)}$$

where *a*,*b*,*c*,…, *j* refer to the content of histidine, isoleucine, leucine, lysine, methionine, phenylalanine, tyrosine, threonine, and valine in each sample, respectively; *ap*, *bp*, *cp*, …, *jp* are the content of histidine, isoleucine, leucine, lysine, methionine, phenylalanine, tyrosine, threonine, and valine in protein standard [28], respectively; *n* is the number of amino acids used.

### 4.6. Mineral Content

The procedure previously described by Lorenzo et al. [82] was used for mineral determination (Ca, K, Mg, Na, P, Fe, Mn, Zn and Cu). For that purpose, a Thermo-Fisher ICAP 6000 plasma emission spectrometer (Thermo-Fisher, Cambridge, UK), equipped with a radio frequency source of 27.12 MHz, a peristaltic pump, a spraying chamber, and a concentric spray nebulizer, totally controlled with a ICP software using 99.996% liquid argon plasma gas (Praxair, Madrid, Spain), was utilized. An external standard for setting the calibration curve was used in order to determine the mineral contents, being the results expressed as mg/100 g.

### 4.7. Statistical Analysis

A total of 140 samples (7 by-products × 2 replicates of each sample × 10 fish pieces) were used to analyse the statistical significance of nutritional differences depending on the fish by-products. Statistical analyses were performed using IBM SPSS Statistics 21 software (IBM Corp., Armonk, NY, USA). Normal distribution and homogeneity of variance were previously tested (Shapiro-Wilk). Data were submitted for analysis of variance (ANOVA) and Duncan test, when ANOVA had a significant effect (*p* < 0.05). The values were given in terms of mean values and standard error of the mean (SEM).

## 5. Conclusions

Research is being carried out to explore the possible use of different fish processing by-products in order to allow their valorisation through the extraction of bioactive compounds, particularly lipids and proteins. More than half of the total wet weight of processed gilthead sea results in processing by-products. These wastes are rich in protein (10%–25%) and fat (17%–35%). Profiles of amino acids, fatty acids, and minerals showed that these discards had high levels of essential amino acids (41%–49% of total amino acids), omega-3 fatty acids (about 13% of total fatty acids), and macro and micro elements, especially calcium and phosphorous in head, fishbone, and gills, and iron and zinc in liver. The results obtained improve the understanding and the characterization of processing by-products from gilthead sea bream, with the aim of ongoing development of value-added products from these wastes. Moreover, the health properties associated with these bioactive compounds give value to these products, resulting in more attractive products for consumers and at the same time avoiding environmental problems linked with these materials. However, more studies are needed on the microbiological evaluation and the safety of these products before they are incorporated as nutraceuticals. Therefore, this study demonstrated the great potential of gilthead sea bream by-products as a promising source of valuable bioactive compounds for the food industry, enabling their use as ingredients in novel foods.

## Figures and Tables

**Table 1:** Dissection percentages of the different parts of gilthead sea bream.

Cuts of Gilthead Sea Bream	Mean	SD
Fins	1.75	0.28
Fishbone	14.57	2.94
Gills	2.63	0.20
Guts	5.04	1.42
Head	21.53	2.65
Heart	0.23	0.11
Liver	1.31	0.28
Muscle	34.12	5.12
Skin	10.25	1.92
Others	3.89	1.05

SD: standard deviation.

**Table 2:** Proximate composition of gilthead sea bream by-products.

Chemical Composition	Muscle	Fishbone	Gills	Guts	Head	Liver	Skin	SEM	Sig.
Moisture	69.07 ^d^	57.64 ^c^	55.24 ^bc^	47.70 ^a^	52.51 ^b^	55.50 ^bc^	45.11 ^a^	0.94	***
Fat	7.86 ^a^	17.13 ^b^	21.55 ^bc^	34.11 ^d^	22.11 ^bc^	25.76 ^c^	26.78 ^c^	1.01	***
Protein	21.05 ^d^	16.39 ^c^	13.92 ^b^	12.89 ^b^	12.91 ^b^	10.11 ^a^	24.78 ^e^	0.49	***
Ash	1.47 ^a^	6.23 ^b^	6.45 ^b^	1.09 ^a^	9.14 ^c^	1.08 ^a^	1.35 ^a^	0.29	***

^a–e^ Means in the same row with different letters differ significantly (*p* < 0.05; Duncan test). SEM: standard error of the mean. Sig.: significance; ***: *p* < 0.001.

**Table 3:** Fatty acid profile of gilthead sea bream by-products (g/100 g of total fatty acids).

Fatty Acid	Muscle	Fishbone	Gills	Guts	Head	Liver	Skin	SEM	Sig.
C14:0	2.38 ^b^	2.84 ^d^	2.62 ^c^	2.64 ^c^	2.79 ^d^	1.83 ^a^	2.61 ^c^	0.04	***
C15:0	0.22 ^b^	0.28 ^d^	0.24 ^c^	0.23 ^c^	0.28 ^d^	0.17 ^a^	0.23 ^c^	0.01	***
C16:0	13.99 ^bc^	13.84 ^bc^	14.13 ^c^	13.27 ^a^	13.99 ^bc^	13.93 ^bc^	13.54 ^ab^	0.07	**
C16:1*n*-7	3.86 ^c^	4.90 ^e^	4.57 ^d^	3.68 ^b^	5.03 ^f^	3.49 ^a^	3.97 ^c^	0.07	***
C17:0	0.18 ^a^	0.55 ^e^	0.19 ^b^	0.20 ^bc^	0.54 ^d^	0.20 ^c^	0.19 ^ab^	0.02	***
C17:1*n*-7	0.16 ^a^	0.45 ^e^	0.20 ^c^	0.15 ^a^	0.43 ^d^	0.18 ^b^	0.16 ^a^	0.02	***
C18:0	2.96 ^bc^	3.07 ^cd^	2.70 ^a^	3.19 ^d^	2.86 ^ab^	4.51 ^e^	2.87 ^ab^	0.07	***
C18:1*n*-9	34.30 ^b^	32.99 ^a^	35.82 ^d^	35.23 ^c^	33.09 ^a^	35.91 ^d^	35.41 ^cd^	0.15	***
C18:1*n*-7	2.63 ^b^	2.72 ^bc^	2.93 ^ef^	2.86 ^de^	2.75 ^cd^	2.98 ^f^	2.39 ^a^	0.03	***
C18:2*n*-6	18.28 ^bc^	18.68 ^cd^	17.82 ^b^	19.58 ^e^	18.23 ^bc^	16.57 ^a^	18.87 ^d^	0.12	***
C20:0	0.27 ^c^	0.27 ^c^	0.28 ^c^	0.30 ^d^	0.25 ^b^	0.18 ^a^	0.28 ^c^	0.01	***
C18:3*n*-6	0.21 ^ab^	0.16 ^a^	0.21 ^ab^	0.21 ^ab^	0.16 ^a^	0.52 ^c^	0.22 ^b^	0.02	***
C20:1*n*-9	2.91 ^c^	1.92 ^a^	2.99 ^cd^	3.06 ^d^	1.90 ^a^	2.13 ^b^	3.08 ^d^	0.06	***
C18:3*n*-3	4.45 ^c^	3.86 ^a^	4.05 ^b^	4.71 ^d^	3.86 ^a^	4.13 ^b^	4.73 ^d^	0.05	***
C20:2*n*-6	0.94 ^a^	0.88 ^a^	0.93 ^a^	0.97 ^a^	0.88 ^a^	1.08 ^b^	0.88 ^a^	0.01	***
C22:1*n*-9	0.57 ^a^	0.65 ^d^	0.58 ^ab^	0.58 ^ab^	0.62 ^cd^	0.57 ^a^	0.60 ^bc^	0.01	***
C20:3*n*-3	0.49 ^d^	0.39 ^a^	0.43 ^b^	0.47 ^c^	0.40 ^a^	0.57 ^e^	0.50 ^d^	0.01	***
C20:4*n*-6	0.39 ^b^	0.45 ^c^	0.31 ^a^	0.28 ^a^	0.46 ^c^	0.50 ^d^	0.28 ^a^	0.01	***
C20:5*n*-3	2.28 ^d^	2.77 ^e^	1.92 ^b^	1.83 ^a^	2.78 ^e^	1.91 ^b^	2.03 ^c^	0.05	***
C24:1*n*-9	0.50 ^bc^	0.43 ^a^	0.52 ^e^	0.49 ^b^	0.41 ^a^	0.52 ^cd^	0.47 ^b^	0.01	***
C22:5*n*-3	1.76 ^d^	2.00 ^e^	1.42 ^a^	1.55 ^b^	2.00 ^e^	2.04 ^e^	1.66 ^c^	0.03	***
C22:6*n*-3	5.20 ^e^	4.58 ^c^	4.09 ^b^	3.51 ^a^	5.00 ^de^	4.90 ^d^	3.98 ^b^	0.08	***
SFA	20.00 ^a^	20.85 ^b^	20.16 ^a^	19.83 ^a^	20.72 ^b^	20.81 ^b^	19.72 ^a^	0.09	***
MUFA	44.92 ^b^	44.05 ^a^	47.61 ^d^	46.05 ^c^	44.23 ^a^	45.77 ^c^	46.08 ^c^	0.15	***
PUFA	34.00 ^d^	33.75 ^cd^	31.17 ^a^	33.10 ^c^	33.77 ^cd^	32.21 ^b^	33.15 ^c^	0.14	***
*n*-3	14.18 ^d^	13.59 ^c^	11.91 ^a^	12.06 ^a^	14.03 ^d^	13.55 ^c^	12.89 ^b^	0.11	***
*n*-6	19.82 ^cd^	20.16 ^cd^	19.26 ^b^	21.04 ^e^	19.73 ^c^	18.66 ^a^	20.26 ^d^	0.10	***
Long chain *n*-3 ^†^	9.23 ^d^	9.35 ^d^	7.43 ^b^	6.89 ^a^	9.78 ^e^	8.86 ^c^	7.67 ^b^	0.13	***
DHA/EPA	2.28 ^e^	1.65 ^a^	2.13 ^d^	1.91 ^c^	1.80 ^b^	2.57 ^f^	1.96 ^c^	0.04	***
*n*-6/*n*-3	1.40 ^a^	1.48 ^b^	1.62 ^c^	1.75 ^d^	1.41 ^a^	1.38 ^a^	1.57 ^c^	0.02	***
PUFA/SFA	1.70 ^d^	1.62 ^bc^	1.55 ^a^	1.67 ^cd^	1.63 ^c^	1.56 ^ab^	1.68 ^cd^	0.01	***

^a–f^ Means in the same row with different letters differ significantly (*p* < 0.05; Duncan test). SEM: standard error of the mean. Sig.: significance; **: *p* < 0.01; ***: *p* < 0.001. ^†^ Long chain *n*-3 PUFA (C20:5*n*-3 +C22:5*n*-3 + C22:6*n*-3). EPA: eicosapentaenoic acid; DHA: docosahexaenoic acid; SFA: saturated fatty acid (C14:0 + C15:0 + C16:0 + C17:0 + C18:0 + C20:0); MUFA: monounsaturated fatty acid (C16:1*n*-7 + C17:1*n*-7 + C18:1*n*-9 + C18:1*n*-7 + C20:1*n*-9 + C22:1n-9 + C24:1*n*-9); PUFA: polyunsaturated fatty acid (C18:2*n*-6 + C18:3*n*-6 + C18:3*n*-3 + C20:2*n*-6 + C20:3*n*-3 + C20:4*n*-6 + C20:5*n*-3 + C22:5*n*-3 + C22:6*n*-3).

**Table 4:** Amino acid content of gilthead sea bream by-products (expressed as mg/100 g of tissue).

Amino Acid	Muscle	Fishbone	Gills	Guts	Head	Liver	Skin	SEM	Sig.
*Non-essential amino acids*									
Aspartic acid	1665.94 ^b^	1490.45 ^b^	873.88 ^a^	707.01 ^a^	974.51 ^a^	579.97 ^a^	1684.35 ^b^	67.50	***
Serine	692.39 ^cd^	745.83 ^d^	500.13 ^ab^	384.69 ^a^	561.69 ^bc^	388.47 ^a^	781.13 ^d^	24.51	***
Glutamic acid	2452.18 ^b^	2152.00 ^b^	1405.68 ^a^	1052.31 ^a^	1504.07 ^a^	1001.47 ^a^	2541.65 ^b^	95.44	***
Glycine	947.23 ^ab^	1647.89 ^c^	1198.47 ^bc^	487.46 ^a^	1582.10 ^bc^	445.77 ^a^	1544.00 ^bc^	81.90	***
Alanine	915.46 ^cd^	1023.35 ^cd^	778.60 ^bc^	449.63 ^a^	825.64 ^bc^	559.99 ^ab^	1156.33 ^d^	38.85	***
Proline	571.67 ^ab^	932.44 ^b^	695.85 ^ab^	354.83 ^a^	883.46 ^b^	439.01 ^a^	948.60 ^b^	44.75	**
Tyrosine	587.79 ^c^	455.09 ^b^	252.92 ^a^	246.60 ^a^	285.10 ^a^	219.13 ^a^	532.17 ^bc^	22.97	***
Total NE	7832.65 ^abc^	8447.04 ^cd^	5705.50 ^ab^	3682.52 ^a^	6616.56 ^bc^	3633.82 ^a^	9188.23 ^d^	313.92	***
*Essential amino acids*									
Histidine	561.41 ^c^	563.80 ^c^	345.73 ^ab^	242.66 ^a^	386.75 ^b^	251.50 ^a^	568.54 ^c^	20.05	***
Arginine	1097.24 ^bc^	1293.64 ^bc^	228.41 ^a^	602.08 ^a^	985.48 ^b^	219.63 ^a^	1456.75 ^c^	63.22	***
Threonine	833.56 ^e^	767.16 ^de^	629.81 ^cd^	413.81 ^ab^	541.25 ^bc^	308.63 ^a^	856.16 ^e^	30.20	***
Valine	914.89 ^b^	803.34 ^b^	547.55 ^a^	498.03 ^a^	514.60 ^a^	566.24 ^a^	924.32 ^b^	32.55	***
Methionine	346.52 ^d^	239.97 ^cd^	n d ^a^	n d ^a^	176.61 ^bc^	117.23 ^b^	285.22 ^c d^	18.24	***
Lysine	1705.13 ^b^	1344.78 ^b^	796.11 ^a^	456.78 ^a^	814.16 ^a^	450.36 ^a^	1646.93 ^b^	76.79	***
Isoleucine	801.69 ^b^	667.67 ^b^	377.97 ^a^	346.84 ^a^	387.14 ^a^	401.80 ^a^	769.59 ^b^	32.14	***
Leucine	1284.54 ^b^	1101.44 ^b^	676.58 ^a^	557.71 ^a^	655.64 ^a^	664.71 ^a^	1290.07 ^b^	51.00	***
Phenylalanine	736.54 ^b^	683.95 ^b^	423.38 ^a^	358.92 ^a^	440.62 ^a^	421.44 ^a^	737.63 ^b^	25.10	***
Total E	8460.81 ^b^	7465.74 ^b^	4025.51 ^a^	3476.82 ^a^	4902.25 ^a^	3401.53 ^a^	8535.21 ^b^	332.90	***
E/NE	1.08 ^c^	0.89 ^b^	0.71 ^a^	0.95 ^b^	0.75 ^a^	0.94 ^b^	0.94 ^b^	0.02	***
Taurine	127.02 ^b^	148.67 ^bc^	n d ^a^	335.20 ^e^	166.53 ^cd^	n d ^a^	187.91 ^d^	10.76	***

^a–e^ Means in the same row with different letters differ significantly (*p* < 0.05; Duncan test). SEM: standard error of the mean. Sig.: significance; **: *p* < 0.01, ***: *p* < 0.001. E: essential amino acids. NE: non-essential amino acids. nd: not detected.

**Table 5:** Nutritional quality of protein of gilthead sea bream by-products.

Amino Acids	FAO/WHO/UNU *	By-Product								
		Muscle	Fishbone	Gills	Guts	Head	Liver	Skin	SEM	Sig.
Histidine	1.5	178.21 ^bc^	229.84 ^e^	165.32 ^abc^	130.30 ^a^	200.32 ^cd^	165.91 ^abc^	153.53 ^ab^	5.90	***
Isoleucine	3.0	127.25 ^bc^	136.34 ^c^	90.43 ^a^	92.97 ^a^	100.19 ^ab^	132.58 ^bc^	103.99 ^abc^	3.84	***
Leucine	5.9	103.68 ^abc^	114.33 ^c^	82.30 ^a^	76.30 ^a^	86.31 ^ab^	111.54 ^bc^	88.64 ^abc^	3.04	**
Lysine	4.5	180.43 ^c^	182.97 ^c^	127.02 ^ab^	82.22 ^a^	140.46 ^bc^	99.10 ^ab^	148.38 ^bc^	6.21	***
Methionine	1.6	103.10 ^b^	91.69 ^b^	nd ^a^	nd ^a^	86.22 ^b^	72.54 ^b^	72.06 ^b^	5.68	***
Phe + Lys	3.8	165.95 ^ab^	183.45 ^b^	127.73 ^a^	128.31 ^a^	148.61 ^ab^	166.79 ^ab^	135.28 ^a^	4.43	***
Threonine	2.3	172.66 ^abc^	204.10 ^c^	196.41 ^c^	144.12 ^ab^	182.80 ^bc^	132.77 ^a^	150.79 ^ab^	5.17	**
Valine	3.9	111.71 ^ab^	126.10 ^bc^	100.74 ^ab^	102.51 ^ab^	102.50 ^ab^	143.68 ^c^	96.04 ^a^	3.13	**
IEAA		138.99 ^bc^	151.67 ^c^	66.73 ^a^	58.63 ^a^	123.51 ^bc^	124.02 ^bc^	114.22 ^b^	4.82	***

^a–e^ Means in the same row with different letters differ significantly (*p* < 0.05; Duncan test). SEM: standard error of the mean. Sig.: significance; **: *p* < 0.01, ***: *p* < 0.001. Phe + Tyr: phenylalanine + tyrosine; IEAA: essential amino acid index; pattern proteins are expressed in (g/100 g protein); nd: not detected. Values of CS and IEAA are from the FAO/WHO/UNU [28] protein pattern *.

**Table 6:** Mineral composition of gilthead sea bream by-products (mg/100 g).

Minerals	Muscle	Fishbone	Gills	Guts	Head	Liver	Skin	SEM	Sig.
*Macro-minerals*									
Calcium	29.11 ^a^	1618.83 ^b^	1873.24 ^c^	19.44 ^a^	2389.24 ^d^	12.81 ^a^	158.60 ^a^	92.72	***
Magnesium	34.68 ^b^	30.70 ^ab^	47.90 ^c^	34.40 ^b^	28.04 ^ab^	49.49 ^c^	26.71 ^a^	1.15	***
Phosphorous	256.38 ^a^	989.20 ^b^	955.92 ^b^	180.51 ^a^	1312.27 ^c^	207.93 ^a^	224.85 ^a^	42.80	***
Potassium	343.45 ^f^	300.67 ^e^	134.95 ^ab^	153.39 ^b^	184.53 ^c^	118.58 ^a^	223.97 ^d^	8.44	***
Sodium	100.00 ^a^	98.00 ^a^	258.80 ^c^	157.93 ^b^	159.30 ^b^	308.79 ^c^	119.24 ^ab^	9.58	***
*Micro-minerals*									
Copper	0.28 ^d^	0.14 ^abc^	0.18 ^bcd^	0.23 ^cd^	0.04 ^a^	0.54 ^e^	0.11 ^ab^	0.02	***
Iron	1.01 ^b^	0.69 ^ab^	2.12 ^c^	1.98 ^c^	0.45 ^a^	3.82 ^d^	0.69 ^ab^	0.11	***
Zinc	0.46 ^a^	1.40 ^b^	2.12 ^d^	1.91 ^cd^	1.71 ^bc^	3.46 ^e^	2.14 ^d^	0.10	***
Manganese ^†^	19.00 ^a^	206.76 ^d^	585.07 ^e^	63.64 ^b^	211.14 ^d^	154.70 ^c^	33.69 ^ab^	16.99	***

^a–f^ Means in the same row with different letters differ significantly (*p* < 0.05; Duncan test). SEM: standard error of the mean. Sig.: significance; ***: *p* < 0.001. ^†^ The manganese contents are expressed as µg/100 g.
